# MultiColor imaging in urticarial vasculitis with recurrent branch retinal artery occlusion in a case with positive toxoplasma IgG and interferon-gamma release assay - Case report

**DOI:** 10.1016/j.ajoc.2022.101437

**Published:** 2022-02-18

**Authors:** Henry Bair, Chun-Ju Lin, You-Ling Li, Ning-Yi Hsia, Chun-Ting Lai, Jane-Ming Lin, Wen-Lu Chen, Chun-Chi Chiang, Yi-Yu Tsai

**Affiliations:** a Department of Ophthalmology, China Medical University Hospital, China Medical University, Taichung, Taiwan; b Stanford University School of Medicine, Stanford, CA, USA; c School of Medicine, College of Medicine, China Medical University, Taichung, Taiwan; d Department of Optometry, Asia University, Taichung, Taiwan

**Keywords:** MultiColor imaging, Recurrent branch retinal artery occlusion, Urticarial vasculitis

## Abstract

**Introduction:**

We report a male who presented with acute visual defect and was diagnosed with urticarial vasculitis with recurrent branch retinal artery occlusion (BRAO) after systemic disease survey, fluorescein angiography (FA), and MultiColor imaging (MCI).

**Case report:**

A 47-year-old male with a history of urticarial vasculitis presented with visual defect OD. Fundus examination showed two foci of ischemic retinal whitening beneath the inferior arcade and above the superior arcade. MCI demonstrated a greenish tinge in the corresponding area. FA revealed segmental arteriolar staining and arterial occlusive changes. BRAO with retinal arteritis was diagnosed. Toxoplasma IgG was positive. Sulfamethoxazole 400mg plus trimethoprim 80mg was given. His vision worsened after 1-week of treatment. The established lesions improved, but new lesions occurred. Interferon-gamma release assay was positive but tuberculosis DNA qualitative amplification test of sputum was negative. Sputum acid-fast stain was positive and culture revealed nontuberculous mycobacteria. Left facial itching and reactive lymphadenopathy developed. Prednisolone and cyclophosphamide were started. The initial retinal artery lesions regained perfusion.

**Conclusions:**

Urticarial vasculitis with recurrent BRAO is an immune complex-mediated disease. Greenish-tinged occlusive lesions were noted from MCI with high resolution and contrast. MCI could be a valuable method for retinal vessel occlusive disease detection before FA and follow up.

## 1 Introduction

Recurrent branch retinal artery occlusion (BRAO) is a rare retinal disease that usually occurs in young patients and is characterized as repeated retinal artery occlusion (RAO) in different focal areas.^1^^,^^2^ Unlike permanent BRAO or transient amaurosis fugax,^3^ the causes of idiopathic BRAO is unclear. It sometimes happens bilaterally and may accompany with vestibuloauditory or sensorimotor symptoms.^1^^,^^2^^,^^4^ The visual prognosis is relatively good. Urticaria with recurrent BRAO is an uncommon syndrome characterized by migraine headaches, tinnitus, vertigo, hearing loss, and recurrent BRAO of unknown etiology.^2^

The MultiColor Module (SPECTRALIS SD-OCT, Heidelberg Engineering, Heidelberg, Germany) is a confocal-scanning laser ophthalmoscopy based imaging system utilizing three monochromatic laser sources simultaneously, including blue (488 nm), green (515 nm), and infrared (820 nm) wavelengths.^5^ Multicolor imaging (MCI) provides diagnostic images that show distinct structures at different depths within the retina and choroid. The information contained in these reflectance images is combined in the MCI and includes structural information from different retinal layers that are visible in the individual reflectance images.

Urticarial vasculitis is an immune complex-mediated disease that has been reported in association with infectious conditions such as hepatitis B and infectious mononucleosis.^6^, ^7^, ^8^ Here we report a male who presented with acute visual defect in the right eye (OD) and who was diagnosed with urticarial vasculitis with recurrent segmental BRAO after a complete systemic disease survey, a series of fluorescein angiography (FA), and MCI. To the best of our knowledge, no published case reports have described MCI findings in this condition.

## 2 Case report

**Figure 1:**
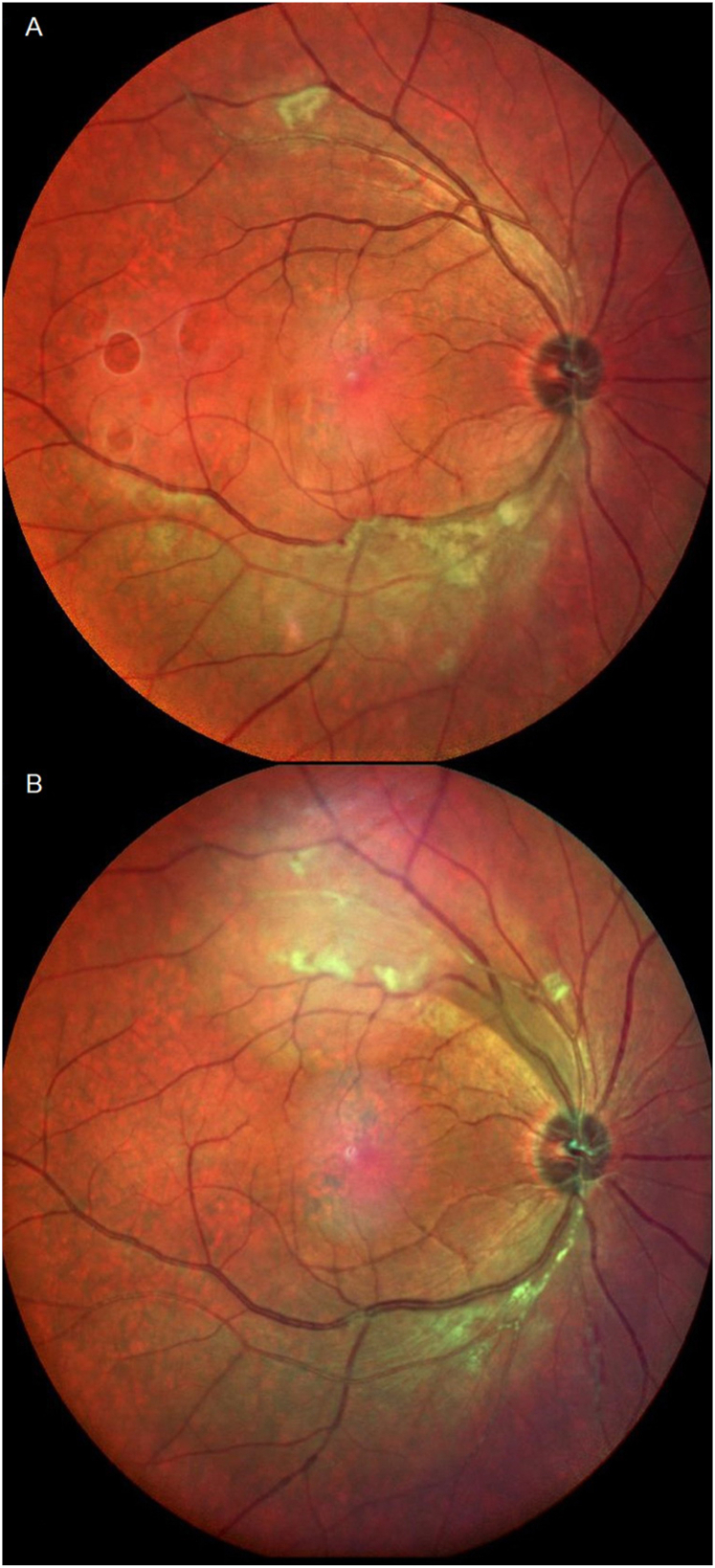
A. MCI demonstrated greenish tinge in the corresponding area indicating intra-retinal thickening. B. MCI demonstrated the two previous lesions improved, but new lesions above the superior arcade occurred.

**Figure 2:**
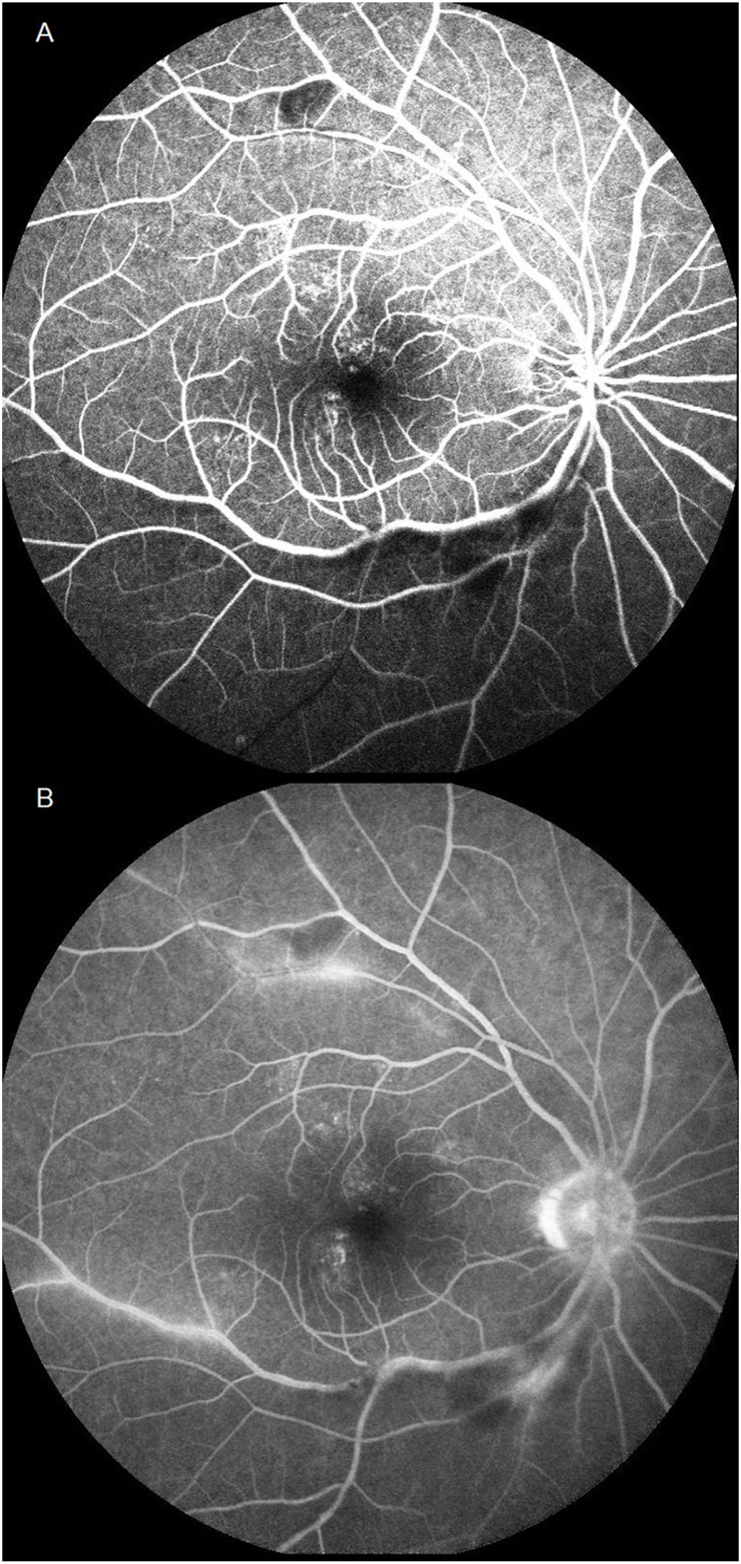
A. FA revealed arterial occlusive changes beneath inferior arcade and above the superior arcade separately. B. FA revealed segmental arteriolar staining in the late phase.

**Fig. 3:**
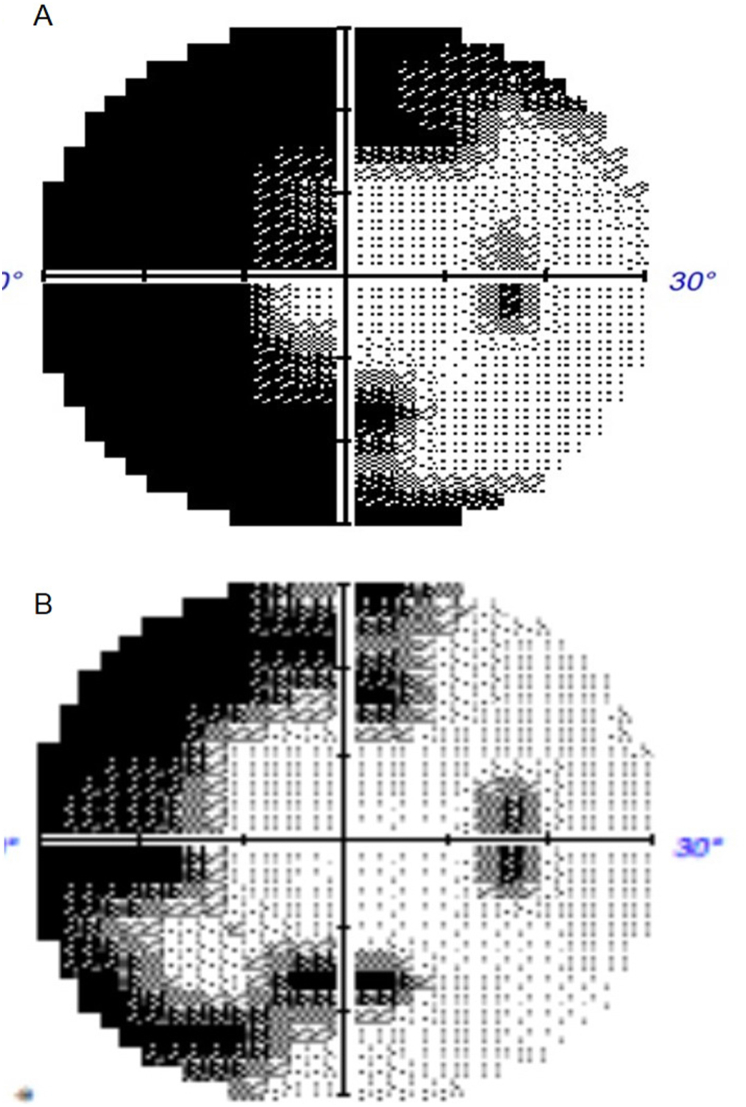
A. VF test showed peripheral field loss. B. VF defect improved after treatment.

The patient, a 47-year-old male, was a smoker with a history of urticarial vasculitis but no significant ocular history. He complained of upper visual defect in the right eye (OD) for three days. His best corrected visual acuity (BCVA) was 20/20 OD and 20/20 in left eye (OS). There were no anterior chamber cells and vitreous haze. Fundus examination showed two foci of ischemic retinal whitening beneath inferior arcade and above the superior arcade separately OD. MCI demonstrated a greenish tinge in the corresponding area, indicating intra-retinal thickening (Fig. 1A). FA revealed segmental arteriolar staining and arterial occlusive changes (Fig. 2A and B, video 1). Visual field (VF) test showed peripheral field loss (Fig. 3A).The fundus was completely normal OS.

These findings were consistent with a diagnosis of BRAO with retinal arteritis, and aspirin and brimonidine were administered. Blood tests were performed and toxoplasma IgG was positive. Although not a typical manifestation, focal retinal vasculitis is one of the findings of ocular toxoplasmosis. Therefore, sulfamethoxazole 400mg plus trimethoprim 80mg was given.

The patient's vision worsened after 1 week of treatment. The two previously identified lesions improved, but new lesions above the superior arcade appeared (Fig. 1B). Meanwhile, interferon-gamma release assay (IGRA) resulted positive. Infectious diseases and pulmonology were consulted. Tuberculosis (TB) DNA qualitative amplification test of sputum was negative. Sputum acid-fast stain was positive and culture only revealed nontuberculous mycobacteria (NTM). Chest X ray was normal, thus chest TB was ruled out.

Since IGRA can react with nontuberculous mycobacterial antigens, a rheumatologist was consulted. Left facial itching and reactive lymphadenopathy were noted. Diagnostic tests for autoimmune diseases, such as systemic lupus erythematosus, Sjogren's syndrome, or other systemic rheumatic diseases, were negative. Urticarial vasculitis with recurrent BRAO was diagnosed. Prednisolone 5 mg and cyclophosphamide 50mg daily were started. Comparing with the previous FA, the initial arterial lesion improved and regained perfusion (video 2). VF defect also improved after treatment (Fig. 3B). His BCVA remained 20/20 for 2 years. The patient tolerated the treatments well and experienced no significant adverse effects.

## 3 Discussion

In idiopathic recurrent BRAO, there is recurrent multifocal BRAOs of unknown etiology and no medical therapy has been shown to be definitively beneficial.^1^^,^^9^ It can develop unilaterally or bilaterally in otherwise healthy individuals who might have encephalopathy, hearing loss, or migraine.^1^^,^^2^^,^^4^ The syndrome consists of multifocal arterial and arteriolar occlusions. Migraine may cause or promote BRAO.^4^ The visual prognosis is relatively better than permanent BRAO.^1^, ^2^, ^3^, ^4^^,^^9^

Chronic urticaria has been reported to accompany various autoimmune or vasculitic diseases.^6^ Urticaria in recurrent BRAO has been reported and may be the result of an immunopathological mechanism.^2^ Affected patients usually undergo a thorough medical workup, however no significant causes of disease are typically found.^2^

Urticarial vasculitis is a clinicopathologic entity typified by recurrent episodes of urticaria that have histopathologic features of leukocytoclastic vasculitis.^7^^,^^8^ Although the majority of cases are of unknown etiology, urticarial vasculitis can be associated with drugs, infections, autoimmune diseases, or malignancy.^7^^,^^8^

Typical manifestations of ocular toxoplasmosis include cells and flares in the anterior chamber, foci of retinitis surrounded by fuzzy retinal edema, vitreous cells, retinal exudates, and focal retinal vasculitis. A significant portion of NTM diseases can result in a positive IGRA. Positive toxoplasma IgG and interferon-gamma release assays in this patient were suspected to be incidental findings.

Another possible diagnosis is Susac syndrome, consisting of a triad including encephalopathy, hearing loss, and BRAO, which occurs mostly in young women. Ophthalmoscopy shows diffuse or localized narrowing of retinal arteries with a “boxcar” segmentation of the blood column at the level of peripheral retinal arteries.^10^ In tubercular retinal vasculitis, another diagnosis considered, periphlebitis is commonly observed and may be accompanied by venous occlusion, peripheral nonperfusion, and eventual neovascularization.^11^ These features, however, were not seen in our patient.

High-resolution, detailed MCI can highlight structures and pathologies not visible on ophthalmoscopy and color fundus photographs (CFP) in lesions of epiretinal membrane,^12^^,^^13^ diabetic macular edema,^14^ and central serous chorioretinopathy.^15^

BRAO induces macular edema in the early stage, and MCI can detect subtle macular edema and retinal thickening as a greenish tinge.^5^ It offers documentation of disease progression and the response to treatment. The color rendering of BRAO on the MCI had a greenish tinge versus the pale area seen on the CFP, which shows the most similarities to fundus examination. Ophthalmologists should appreciate that the MCI might differ from what is observed clinically.^5^

In this case, obvious ischemic lesions with greenish tinges were demonstrated by MCI, which has higher resolution and contrast than conventional CFP. MCI clearly showed that while the previous BRAO lesion beneath the inferior arcade had improved, a new lesion above the superior arcade occurred.

The visual prognosis of urticarial vasculitis with recurrent segmental BRAO can be relatively good. Greenish-tinged occlusive lesions were noted from MCI with high resolution and contrast. MCI is a valuable method for retinal vessel occlusion disease detection before FA and follow up. Clinicians should be aware of the differences in the appearance of BRAO lesions between MCI and CFP.

## Declarations

Ethics approval and consent to participate.

The need for approval was waived due to deidentification.

## Consent for publication

Written informed consent for publication of his clinical details and clinical images was obtained from the patient.

## Availability of data and material

All data generated or analyzed during this study are included in this published article.

## Conflicts of interest

The authors declare that they have no competing interests.

## Funding

No.

## Authors' contributions

HB, CJL, YLL, NYH, CTL, and YYT were responsible for substantial contributions to the conception or design of the work, and acquisition of data. HB and CJL were responsible for interpretation of results. HB and CJL participated in the design and was a major contributor in writing the manuscript. HB, CJL, YLL, NYH, CTL, JML, WLC, WCW, and YYT were responsible for final approval of the version to be published. All authors have read and approved the manuscript.

## Ethics statement

We obtained the written informed consent to use the patient's information for the publication of this paper.

## Declaration of competing interest

The authors declare that the research was conducted in the absence of any commercial or financial relationships that could be construed as a potential conflict of interest.

## List of abbreviations

BRAO: branch retinal artery occlusion
FA: fluorescein angiography
MCI: multicolor imaging
IGRA: interferon-gamma release assay
TB: tuberculosis
VF: visual field
NTM: nontuberculous mycobacteria
CFP: color fundus photograph

## Appendix A Supplementary data

Video 1The initial FA revealed segmental arteriolar staining and arterial occlusive changes.

Video 2Comparing with the previous FA, the initial arterial lesion improved and regained perfusion.
